# Coroner’s Inquest

**Published:** 1855-02

**Authors:** 


Most of the New York Medical Journals have recently given some account of a coroner's inquest, held in Brooklyn, over the body of Agnes E. Lottimer, a child who had been homœopathically victimized.

The hot stage of an intermittent, mistaken for phrenitis, was treated by aconite and belladonna in the thirtieth dilution.

"Suffice it to say, the patient got worse and worse; passed from the 'inflammation of the brain' through 'ague and fever without an intermission,' 'regular intermittent' complicated with *pruritus*, (all the time getting well) 'up to the three days before her death,' when it became complicated with mumps, which was translated to the membranes of the brain.' The 'mumps,' in this instance, presented the peculiarity of not involving the parotid gland; the ague and fever of being unintermitted; and the death which rapidly ensued, in full harmony with the other singularities of the case, was ultimately induced 'by suffocation from the accumulation of blood in the throat,' said blood having found its way there out of the longitudinal sinus, which having been ruptured poured its contents *through the cribriform plate of the ethmoid bone!*"

A more melancholy instance of the wretched ignorance and folly of quackery, has rarely been witnessed. It is not easy to be charitable toward such malpractice, and Tyburn tree, hung with homœopaths as thick as apples in September, becomes a pleasant prospect. H.

